# Genomic analysis of 40 prophages located in the genomes of 16 carbapenemase-producing clinical strains of *Klebsiella pneumoniae*

**DOI:** 10.1099/mgen.0.000369

**Published:** 2020-04-29

**Authors:** Ines Bleriot, Rocío Trastoy, Lucia Blasco, Felipe Fernández-Cuenca, Antón Ambroa, Laura Fernández-García, Olga Pacios, Elena Perez-Nadales, Julian Torre-Cisneros, Jesús Oteo-Iglesias, Ferran Navarro, Elisenda Miró, Alvaro Pascual, German Bou, Luis Martínez-Martínez, Maria Tomas

**Affiliations:** ^1^​ Microbiology Department, Research Institute Biomedical A Coruña (INIBIC), Hospital A Coruña (CHUAC), University of A Coruña (UDC), A Coruña, Spain; ^2^​ Study Group on Mechanisms of Action and Resistance to Antimicrobials (GEMARA), Spanish Society of Infectious Diseases and Clinical Microbiology (SEIMC), Madrid; ^3^​ Clinical Unit for Infectious Diseases, Microbiology and Preventive Medicine, Hospital Universitario Virgen Macarena. Deparment of Microbiology and Medicine, University of Seville, Seville, Spain; ^4^​ Spanish Network for the Research in Infectious Diseases, REIPI, Seville, Spain; ^5^​ Microbiology Unit, Maimonides Biomedical Research Institute of Cordoba (IMIBIC), University Hospital Reina Sofía, University of Córdoba, Cordoba, Spain; ^6^​ Reference and Research Laboratory for Antibiotic Resistance and Health Care Infections, National Centre for Microbiology, Institute of Health Carlos III, Majadahonda, Madrid, Spain; ^7^​ Microbiology Department, Sant Pau Hospital, Autonomous University of Barcelona (Bellaterra), Barcelona, Spain

**Keywords:** *Klebsiella pneumoniae*, prophages, bioinformatics, genomic analysis, comparative genomics, phylogeny

## Abstract

*
Klebsiella pneumoniae* is the clinically most important species within the genus *Klebsiella* and, as a result of the continuous emergence of multi-drug resistant (MDR) strains, the cause of severe nosocomial infections. The decline in the effectiveness of antibiotic treatments for infections caused by MDR bacteria has generated particular interest in the study of bacteriophages. In this study, we characterized a total of 40 temperate bacteriophages (prophages) with a genome range of 11.454–84.199 kb, predicted from 16 carbapenemase-producing clinical strains of *K. pneumoniae* belonging to different sequence types, previously identified by multilocus sequence typing. These prophages were grouped into the three families in the order *Caudovirales* (27 prophages belonging to the family *Myoviridae*, 10 prophages belonging to the family *Siphoviridae* and 3 prophages belonging to the family *Podoviridae*). Genomic comparison of the 40 prophage genomes led to the identification of four prophages isolated from different strains and of genome sizes of around 33.3, 36.1, 39.6 and 42.6 kb. These prophages showed sequence similarities (query cover >90 %, identity >99.9 %) with international Microbe Versus Phage (MVP) (http://mvp.medgenius.info/home) clusters 4762, 4901, 3499 and 4280, respectively. Phylogenetic analysis revealed the evolutionary proximity among the members of the four groups of the most frequently identified prophages in the bacterial genomes studied (33.3, 36.1, 39.6 and 42.6 kb), with bootstrap values of 100 %. This allowed the prophages to be classified into three clusters: A, B and C. Interestingly, these temperate bacteriophages did not infect the highest number of strains as indicated by a host-range assay, these results could be explained by the development of superinfection exclusion mechanisms. In addition, bioinformatic analysis of the 40 identified prophages revealed the presence of 2363 proteins. In total, 59.7 % of the proteins identified had a predicted function, mainly involving viral structure, transcription, replication and regulation (lysogenic/lysis). Interestingly, some proteins had putative functions associated with bacterial virulence (toxin expression and efflux pump regulators), phage defence profiles such as toxin–antitoxin modules, an anti-CRISPR/Cas9 protein, TerB protein (from ter*ZABCDE* operon) and methyltransferase proteins.

## Data Summary

The genome sequences of the temperate bacteriophages (prophages) of the clinical strains of *Klebsiella pneumoniae* (Spanish Network for Research in Infectious Diseases, REIPI) have been deposited in GenBank under BioProject accession number PRJNA565865 (http://www.ncbi.nlm.nih.gov/bioproject/565865). The genome and ORF data for the bacteriophages are included in this BioProject record.

**Impact Statement**

*
Klebsiella pneumoniae* is a successful multi-drug resistant human pathogen and an important source of hospital infections associated with high morbidity and mortality due to multiple factors. Temperate bacteriophages (prophages), located in the genome of clinical strains of *K. pneumoniae *, act as mobile elements of horizontal gene transfer, being able to modulate the behaviour of bacteria by providing virulence and phage defence genes. Therefore, the characterization of these prophages can lead to the discovery of new molecular targets for the development of innovative antibacterial treatments.

## Introduction

Bacteriophages, i.e. viruses that infect bacteria, are the most abundant biological entities on Earth [1–3]. They are found in all environmental niches colonized by bacteria, with an estimated global population of 10^31^ viral particles [4, 5]. Bacteriophages have very diverse genomes and have been suggested to represent the largest source of gene diversity in the environment, as highlighted by the large number of novel genes of unknown function revealed by genome and metavirome sequencing [6]. Detailed comparative analysis has been made of the bacteriophages that infect various hosts and environments, including *Mycobacterium* [7], *Acinetobacter* [8]*, Pseudomonas* [9], *Bacillus* [10], *Lactococcus* [11], marine cyanobacterium [12], *Salmonella* [13] and *Vibrio* species [14] and also members of the family *Enterobacteriaceae* [15]. However, the analysis of the families of all sequenced bacteriophages has illustrated how little of the global phage population has been genomically sampled [16, 17]. With an almost endless supply of diverse phages readily accessible for isolation and analysis, research programmes will continue to play substantial roles in analysing and describing functional proteins in the phages to understand their ecology and the various clinical, industrial and biotechnological applications [18].

Genomic comparative analysis has highlighted the mosaicism present in the genomes of all the dsDNA tailed bacteriophages. The term mosaicism refers to the fact that bacteriophages harbour in their genomes different regions with different evolutionary history due to the phenomenon of horizontal gene transfer (HGT) [16, 17]. The phylogeny and evolutionary relationships between bacteriophages isolated from bacteria have been analysed in several studies [19, 20], confirming their importance. The phenomenon of HGT, as well as bacteriophages themselves, act as drivers of the evolution and diversification of bacteria, allowing them to acquire virulence factors (e.g. diphtheria or botulinum toxins) and/or genes related to metabolism, antibiotic resistance (e.g. β-lactamases) and adaptation to new environmental niches [21–23].

*Klebsiella pneumoniae,* which belongs to the family *Enterobacteriaceae *, is a Gram-negative opportunistic bacterial pathogen associated with a wide range of diseases, such as urinary tract infections, pneumonia and septicaemia, as well as infection of wounds and soft tissues [24]. In recent years, different strains of this species have acquired genes for resistance to antibiotics, especially genes that encode enzymes capable of breaking down most β-lactamases [25]. This has led to the generation of multi-drug resistant (MDR) pathogens, which constitute a serious public-health problem [26]. The success of *K. pneumoniae* as a nosocomial pathogen is due to its intrinsic virulence, attributed to its ability to cause invasive infection via fimbrial adhesins [27] and to the presence of a thick capsule, which acts as a possible antiphagocytic factor [28], and to toxin–antitoxin (TA) systems associated with the stability of mobile elements acquired through HGT, such as *vagCD* genes, which encode a functional broad-spectrum TA system and are conserved on the large multiple-antibiotic-resistance-conferring plasmids in this species [29].

In this study, we analysed the genomic characteristics of 40 genomes of prophages identified in 16 clinical isolates of carbapenem-producing *K. pneumoniae *. Moreover, we used comparative bioinformatic tools and microscopic techniques to investigate the phylogeny and evolutionary relationships of the prophages.

## Methods

### Origin of the *K. pneumoniae* genomes

In this study, we analysed 16 isolates of *K. pneumoniae* belonging to different sequence types (STs), previously determined by multilocus sequence typing (MLST), isolated from clinical samples and harbouring the most prevalent carbapenemase genes (OXA 48, VIM and KPC β-lactamases) (Table 1). The STs and carbapenemase β-lactamases were previously determined by Esteban-Cantos *et al*., according to the methods proposed by the Pasteur Institute (https://bigsdb.pasteur.fr/klebsiella/klebsiella.html) [30]. The study highlighted that *K. pneumoniae* ST258 and ST15 are high-risk clones in the worldwide spread of carbapenemases.

**Table 1.** Characteristics of the *K. pneumoniae* clinical strains and their origins (all isolated in Spain between November 2013 and April 2014)

Strain	MLST*	Carbapenemase	Origin	GenBank accession no.†	Genome size (bp)	G+C (mol%)
ST405-OXA48	ST-405	OXA48	Wound	WRXJ00000000	5 774 094	57.0
ST15-VIM1	ST-15	VIM1	Blood	WRXI00000000	5 122 717	57.0
ST11-OXA245	ST-11	OXA245	Wound	WRXH00000000	5 667 817	57.1
ST437-OXA245	ST-437	OXA245	Rectal	WRXG00000000	5 653 384	57.2
ST16-OXA48	ST-16	OXA48	Urine	WRXF00000000	5 447 971	57.3
ST101-KPC2	ST-101	KPC2	Rectal	WRXE00000000	5 523 997	57.2
ST147-VIM1	ST-147	VIM1	Rectal	WRXD00000000	5 695 918	56.8
ST11-VIM1	ST-11	VIM1	Respiratory	WRXC00000000	5 594 690	57.2
ST846-OXA48	ST-846	OXA48	Sputum	WRXB00000000	5 548 215	57.3
ST340-VIM1	ST-340	VIM1	Rectal	WRXA00000000	5 539 743	57.2
ST13-OXA48	ST-13	OXA48	Rectal	WRWZ00000000	5 585 239	57.0
ST512-KPC3	ST-512	KPC3	Axillary smear	WRWY00000000	5 650 413	57.2
ST15-OXA48	ST-15	OXA48	Axillary smear	WRWX00000000	5 188 264	57.3
ST11-OXA48	ST-11	OXA48	Urine	WRWW00000000	5 509 234	57.3
ST258-KPC3	ST-258	KPC3	Urine	WRWV00000000	5 564 025	57.3
ST974-OXA48	ST-974	OXA48	Urine	WRWT00000000	5 414 512	57.3

*Institute Pasteur MLST database (https://pubmlst.org/kpneumoniae).

†GenBank accession numbers for the genomes of the 16 *K. pneumoniae* clinical strains, all belonging to BioProject accession number PRJNA565865 (https://www.ncbi.nlm.nih.gov/bioproject/?term=PRJNA565865).

Genomic DNA was isolated from the strains with a Wizard genomic DNA kit (Promega), following the manufacturer’s protocol for extraction, for subsequent sequencing of the different genomes with the MiSeq system (Illumina). Sequences of 250 bp paired-end reads of each isolate were assembled ‘*de novo*’ with Velvet v.1.2.10 (https://www.ebi.ac.uk/~zerbino/velvet/). All the assembly attributes were included in GenBank BioProject PRJNA565865 (https://www.ncbi.nlm.nih.gov/bioproject/?term=PRJNA565865).

### Identification of prophages in the genome of *K. pneumoniae* isolates

Prophages in the genomes of different strains of *K. pneumoniae* were identified with the phaster (Phage Search Tool –Enhanced Release) bioinformatics tool (http://phaster.ca/). Only those temperate bacteriophages identified by the program as intact (score >90) were used for analysis in the present study.

The comparative genomic analysis of the four *K. pneumoniae* prophages found in different isolates of *K. pneumoniae* was carried out with the blast Ring Image Generator (brig) program [31], which uses the sequence similarity between the bacteriophage regions and the sequences of the assembled bacterial genome. Focusing on bacteriophage clusters of the Microbe Versus Phage (MVP) database (http://mvp.medgenius.info) in *K. pneumoniae* strains, we searched for possible homologies between these groups and the prophages under study. For this purpose, we used the Position-Specific Iterative Basic Local Alignment Search Tool (psi-blast) to analyse the complete prophage genome against the representative sequence (i.e. the longest) of the cluster and applied the following cut off values: query coverage >60 %, identity >89 %.

The MVP database can be used to study phage–host relationships [32], as it provides the user with an important list of interactions in addition to international clusters (sets of viral sequences grouped according to their sequence similarities). We searched the database for possible sequence homologies between the 40 prophages under study and the international clusters. We observed homology with 25 international clusters of *K. pneumoniae* (549, 934, 1013, 1319, 1690, 1920, 2329, 2808, 3207, 3429, 3546, 3969, 4280, 4331, 4575, 4762, 4864, 4868, 4901, 5263, 8737, 9608, 9912, 10255, 11441), by applying the aforementioned cut-off values.

### Integration of prophages

In the final step of the study, the integration sites of the prophages were identified with the Standard Nucleotide Basic Local Alignment Search Tool (blastn) by searching for nucleotide sequence similarity between the *K. pneumoniae* prophages and all sequences in the National Center for Biotechnology Information (NCBI) database. Groups of proteins located before the integrase proteins were considered integration sites.

### Phylogenic relationships among the 40 temperate bacteriophages predicted to belong to the order *Caudovirales*

The dot plot alignment of the nucleotide sequences of the 40 temperate bacteriophages was inferred using Genome Pair Rapid Dotter (gepard) [33], and a fasta file was constructed with all the sequences. Bacteriophages lack a universal marker gene for phylogenetic analysis; however, the use of the terminase large subunit of the tail protein or the major capsid protein is often reported [34]. In the present study, a phylogenetic analysis of the sequences of the terminase large subunit was performed with the ClustalW program (http://www.ebi.ac.uk/clustalw/) in mega x software [35]. A tree was generated by multiple alignments, with the neighbour-joining method. The following parameters were applied to produce the tree: (i) the number of differences was established; (ii) gaps/missing data were treated as complete deletions; (iii) the bootstrap consensus tree was inferred from 1000 replicates [36]; and (iv) the condensed tree was displayed with a value of 100 %.

### Isolation of the bacteriophages and transmission electron microscopy (TEM) studies

Mitomycin C was used to induce bacteriophage production following the protocol described by López *et al*. [8]. For this purpose, 15 ml Luria–Bertani (LB) broth was inoculated with 150 µl overnight culture of strains and incubated with shaking (180 r.p.m.) at 37 °C, until the optical density measured at wavelength 600 nm (OD_600_) reached 0.5. Mitomycin C was then added (10 µg ml^−1^) and the culture was allowed to grow, with shaking (180 r.p.m.) at 37 °C, until lysis occurred, i.e. when the culture appeared clear (after approximately 2–4 h). The lysates were centrifuged at 3500 r.p.m. for 10 min, and the supernatant was filtered through a 0.22 nm filter (Millipore Express PES membrane; Merck). NaCl was added (to a final concentration of 0.5 M), and the suspensions were then mixed and left on ice for 1 h. The suspensions were centrifuged at 3500 r.p.m. for 40 min at 4 °C, and the supernatants were transferred to sterile tubes. PEG 6000 (10 %, w/v) was added and dissolved by rocking the tubes at room temperature for 1 h and incubating them overnight at 4 °C. Bacteriophages were then precipitated at 3500 r.p.m. for 40 min at 4 °C and resuspended in SM buffer (0.1 M NaCl, 1 mM MgSO_4_, 0.2 M Tris-HCl, pH 7.5) [37]. Finally, the samples were stored at 4 °C until processed for TEM on a JEOL JEM-1011 electron microscope.

### Induced bacteriophage mix spot test

Spot tests were carried out using the mixed induced samples of bacteriophages obtained from the purified PEG preparation used for TEM. All of them contained representative members of each cluster (A, B, C, D and E). The spot test method was a modified version of the protocol described by Raya and Hebert [38]. When the OD_600_ reached 0.6 nm, 200 µl culture from the 16 *K *. *pneumoniae* isolates was mixed with 4.5 ml soft agar (0.5 % NaCl, 1 % tryptone and 0.4 % agar) and poured onto TA agar plates (0.5 % NaCl, 1 % tryptone and 1.5 % agar). Once the soft medium had solidified, 15 µl bacteriophage mix solutions were added to the TA agar plates. A negative control consisting of SM buffer was included for each plate.

### ORF annotation

The putative functions of the ORFs were determined by sequence identity with the Rapid Annotation by Subsystem Technology (rast) server (http://rast.nmpdr.org/) and the blast-protein tool psi-blast developed by the NCBI (https://blast.ncbi.nlm.nih.gov/Blast.cgi). In addition, we used the HHpred tool in the MPI informatics toolkit (https://toolkit.tuebingen.mpg.de/#hhpred/) [39], which predicts functions through protein structure with a model accuracy that is competitive with that of the best servers in CASP8. The cut-off *E* value for annotation of proteins was <10^−5^ and all ORFs with *E* values >10^5^ were annotated as hypothetical proteins (Critical Assessment of Technique for Protein Structure Prediction) [40].

## Results

### Predicted prophage genomes

Whole-genome sequencing of the 16 carbapenemase-producing clinical strains of *K. pneumoniae* (Table 2) and the use of the phaster bioinformatics tool (http://phaster.ca/) revealed the presence of a total of 40 prophages considered intact (score >90), all belonging to the order *Caudovirales*. Most were members of the family *Myoviridae* (27 prophages), and the others belonged to the families *Siphoviridae* (10 prophages) and *Podoviridae* (3 prophages). The families were assigned by sequence homologies with the most common bacteriophage indicated by phaster in the Virus-Host Database (https://www.genome.jp/virushostdb/).

**Table 2.** Characteristics of the genome sequences of the 40 prophages found in 16 clinical strains of carbapenemase-producing *K. pneumoniae* (BioProject accession number PRJNA565865; https://www.ncbi.nlm.nih.gov/bioproject/?term=PRJNA565865)

Bacteriophage	Family	Accession no.	Genome size (bp)	No. of ORFs	Hypothetical protein (%)	G+C (mol%)	tRNA
*** Klebsiella pneumoniae* ST405-OXA48**							
ST405-OXA48phi1.1	*Myoviridae*	MK388859.1	35.232	52	48.1	53.8	0
ST405-OXA48phi1.2	*Myoviridae*	MK416007.1	40.495	67	59.7	52.1	0
ST405-OXA48phi1.3	*Siphoviridae*	MK416008.1	32.010	47	66.0	50.9	0
*** Klebsiella pneumoniae* ST15-VIM1**							
ST15VIM1phi2	*Myoviridae*	MK448228.1	46.342	73	54.8	53.6	0
*** Klebsiella pneumoniae* ST11-OXA245**							
ST11-OXA245phi3.1	*Myoviridae*	MK416009.1	33.326	44	20.5	51.5	0
ST11-OXA245phi3.2	*Podoviridae*	MK416010.1	60.118	72	37.5	55.6	0
*** Klebsiella pneumoniae* ST437-OXA245**							
ST437-OXA245phi4.1	*Myoviridae*	MK416011.1	39.642	53	26.4	52.8	0
ST437-OXA245phi4.2	*Myoviridae*	MK416012.1	18.281	27	25.9	50.5	0
*** Klebsiella pneumoniae* ST16-OXA48**							
ST16-OXA48phi5.1	*Siphoviridae*	MK416013.1	57.025	80	50.0	53.6	0
ST16-OXA48phi5.2	*Myoviridae*	MK448230.1	47.305	79	54.8	53.8	4
ST16-OXA48phi5.3	*Siphoviridae*	MK416014.1	29.301	51	49.0	50.2	0
ST16-OXA48phi5.4	*Myoviridae*	MK416015.1	38.322	46	28.3	50.1	0
*** Klebsiella pneumoniae* ST101-KPC2**							
ST101-KPC2phi6.1	*Myoviridae*	MK448231.1	48.131	75	53.2	52.6	4
ST101-KPC2phi6.2	*Myoviridae*	MK416016.1	11.454	17	11.8	59.0	0
ST101-KPC2phi6.3	*Siphoviridae*	MK416017.1	43.942	62	43.1	52.2	3
*** Klebsiella pneumoniae* ST147-VIM1**							
ST147-VIM1phi7.1	*Myoviridae*	MK416018.1	34.141	43	20.5	53.0	1
ST147-VIM1phi7.2	*Podoviridae*	MK448232.1	34.200	45	38.6	40.0	0
*** Klebsiella pneumoniae* ST11-VIM1**							
ST11-VIM1phi8.1	*Myoviridae*	MK448233.1	42.666	67	37.0	50.7	2
ST11-VIM1phi8.2	*Podoviridae*	MK448234.1	48.230	68	41.7	52.9	0
ST11-VIM1phi8.3	*Myoviridae*	MK416019.1	39.953	62	45.1	51.6	0
ST11-VIM1phi8.4	*Myoviridae*	MK416020.1	33.016	43	20.9	51.6	0
*** Klebsiella pneumoniae* ST846-OXA48**							
ST846-OXA48phi9.1	*Siphoviridae*	MK416021.1	38.370	38	36.8	53.3	0
ST846-OXA48phi9.2	*Myoviridae*	MK416022.1	57.402	91	63.7	51.6	0
*** Klebsiella pneumoniae* ST340-VIM15**							
ST340-VIM1phi10.1	*Myoviridae*	MK422455.1	36.124	58	58.6	51.7	0
ST340-VIMphi10.2	*Myoviridae*	MK422454.1	33.326	45	24.4	51.5	0
*** Klebsiella pneumoniae* ST13-OXA48**							
ST13-OXA48phi12.1	*Myoviridae*	MK422453.1	39.086	50	19.6	53.5	1
ST13-OXA48phi12.2	*Siphoviridae*	MK422452.1	34.141	51	49.0	50.5	0
ST13-OXA48phi12.3	*Siphoviridae*	MK422451.1	84.199	93	47.7	49.9	0
ST13-OXA48phi12.4	*Siphoviridae*	MK422450.1	59.049	86	57.5	52.1	0
ST13-OXA48phi12.5	*Siphoviridae*	MK714353.1	44.913	72	58.9	51.8	2
*** Klebsiella pneumoniae* ST512-KPC3**							
ST512-KPC3phi13.1	*Myoviridae*	MK448235.1	42.666	67	47.8	52.9	2
ST512-KPC3phi13.2	*Myoviridae*	MK422449.1	32.302	44	25.0	51.9	0
ST512-KPC3phi13.5	*Myoviridae*	MN166823.1	25.624	45	55.6	52.0	0
ST512-KPC3phi13.6	*Myoviridae*	MK433577.1	39.643	52	28.8	52.8	0
*** Klebsiella pneumoniae* ST15-OXA48**							
ST15-OXA48phi14	*Myoviridae*	MK433578.1	33.839	47	21.3	52.7	0
*** Klebsiella pneumoniae* ST11-OXA48**							
ST11-OXA48phi15.1	*Myoviridae*	MK433579.1	36.137	58	55.2	51.7	0
ST11-OXA48phi15.3	*Myoviridae*	MK433580.1	33.326	44	20.5	51.5	0
*** Klebsiella pneumoniae* ST258-KPC3**							
ST258-KPC3phi16.1	*Myoviridae*	MK433581.1	39.643	53	32.1	52.8	0
ST258-KPC3phi16.2	*Myoviridae*	MK433582.1	33.326	45	22.2	51.5	0
*** Klebsiella pneumoniae* ST974-OXA48**							
ST974-OXA48phi18	*Siphoviridae*	MK448237.1	51.967	80	54.3	52.9	1

**Fig. 1.**
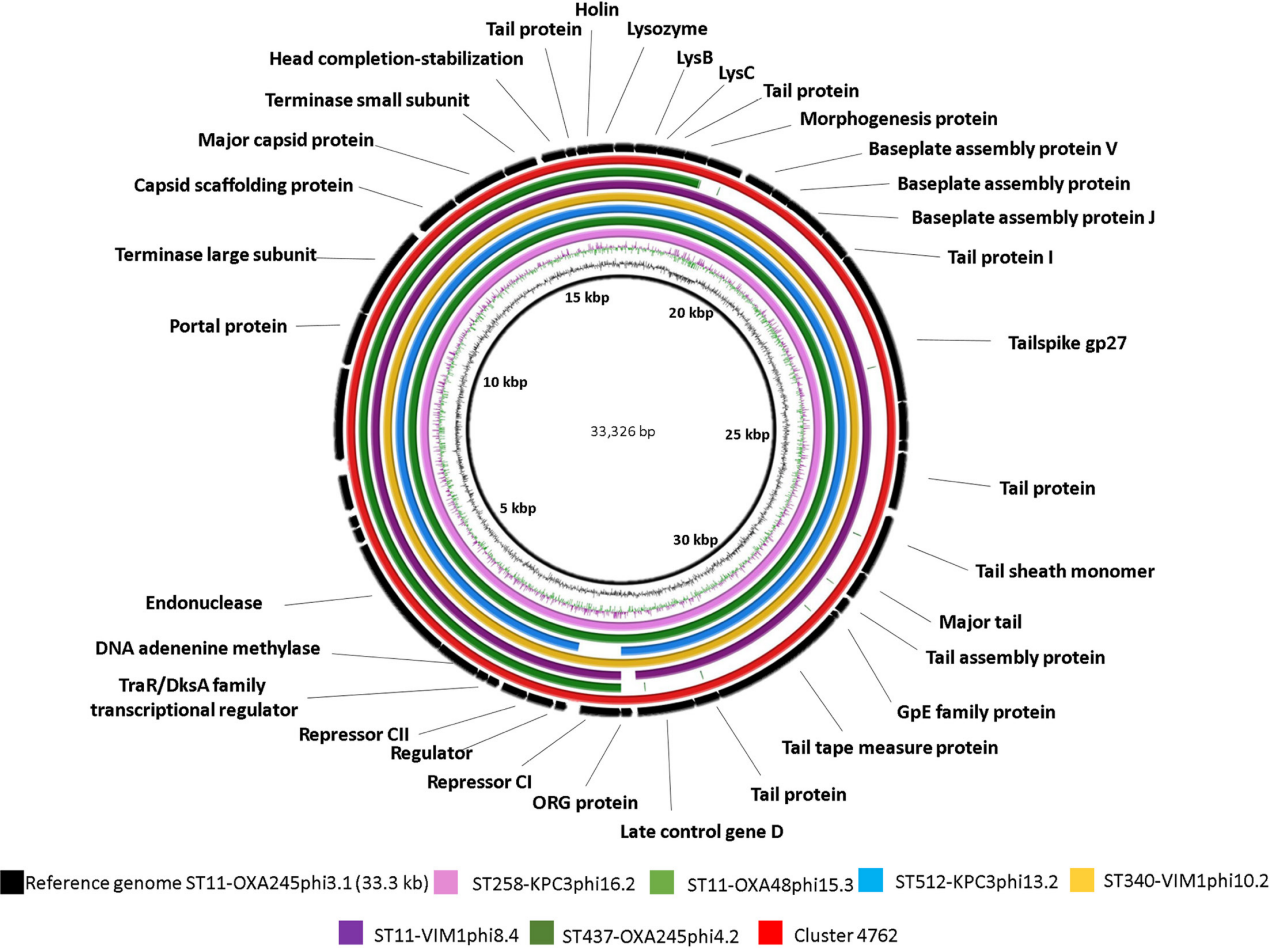
Comparative genomic analysis of *K. pneumoniae* temperate bacteriophages displaying the highest sequence identity with cluster 4762 (query cover >99.9 %; identity >99.8 %) from the MVP database (https://mvp.medgenius.info), constructed with the brig program. The sequence (ST11-OXA245phi3.1) is located on the innermost side and is indicated in black. The double ring adjacent to the reference sequence represents the G+C content (black) and the G+C skew (purple and green). The other rings are as indicated in the key. The white parts of the rings represent absent or divergent content relative to the unknown sequence. Most of the prophages were approximately 33.3 kb in size (ST11-VIM1phi8.4, ST340-VIM1phi10.2, ST512-KPC3phi13.2, ST11-OXA48phi15.3 and ST258-KPC3phi16.2).

**Fig. 2.**
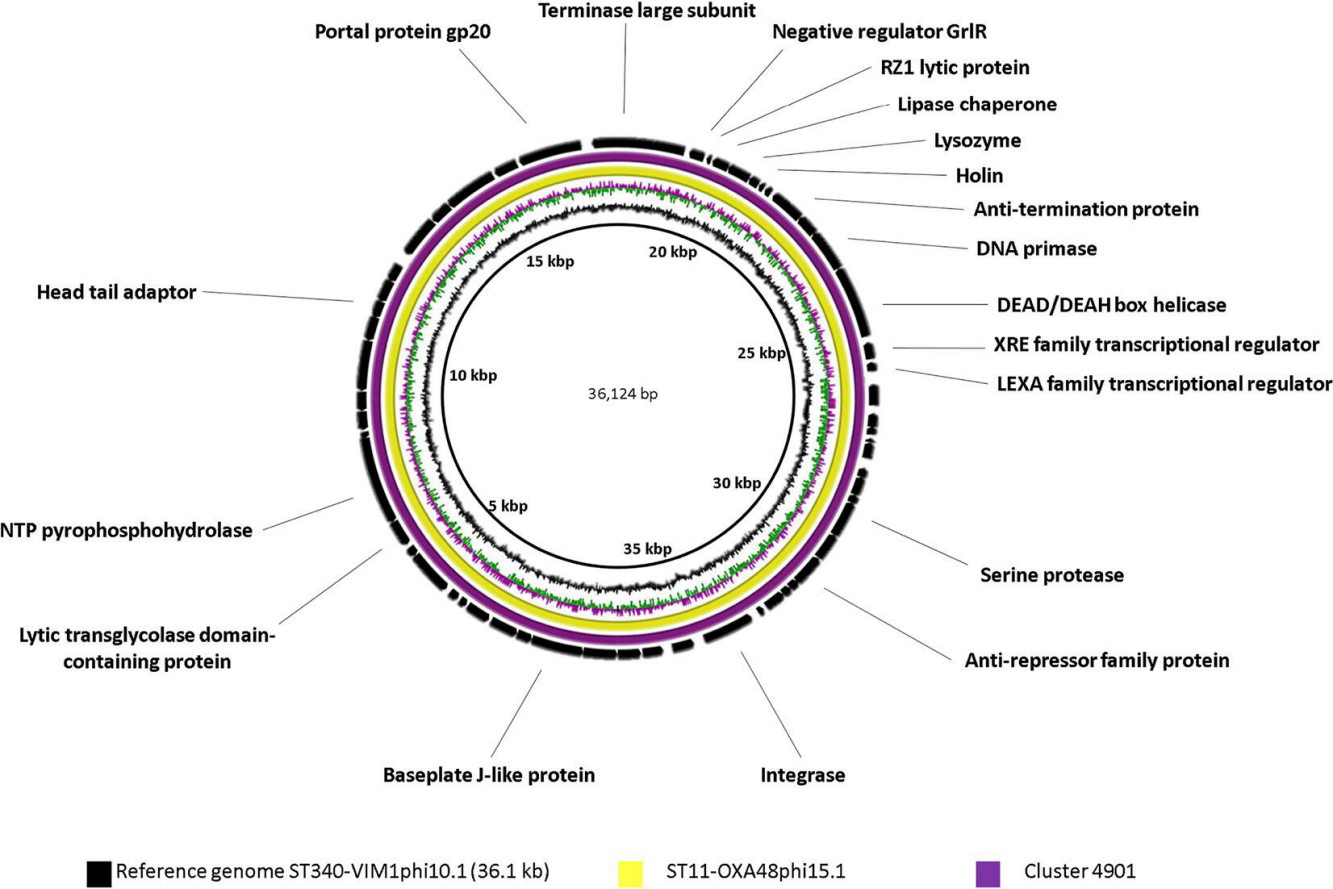
Comparative genomic analysis of *K. pneumoniae* temperate bacteriophages displaying the highest sequence identity with cluster 4901 (% query cover >98.9; % identity >99.9) of the MVP database [https://mvp.medgenius.info], constructed with the BRIG program. The sequence (ST340-VIM1phi10.1) is located on the innermost side and is indicated in black. The double ring adjacent to the reference sequence represents the GC content (black) and the GC skew (purple and green). The white part of the rings represents absent or divergent content relative to the unknown sequence. All prophages were of size 36.1 kb (ST11-OXA48phi15.1).

**Fig. 3.**
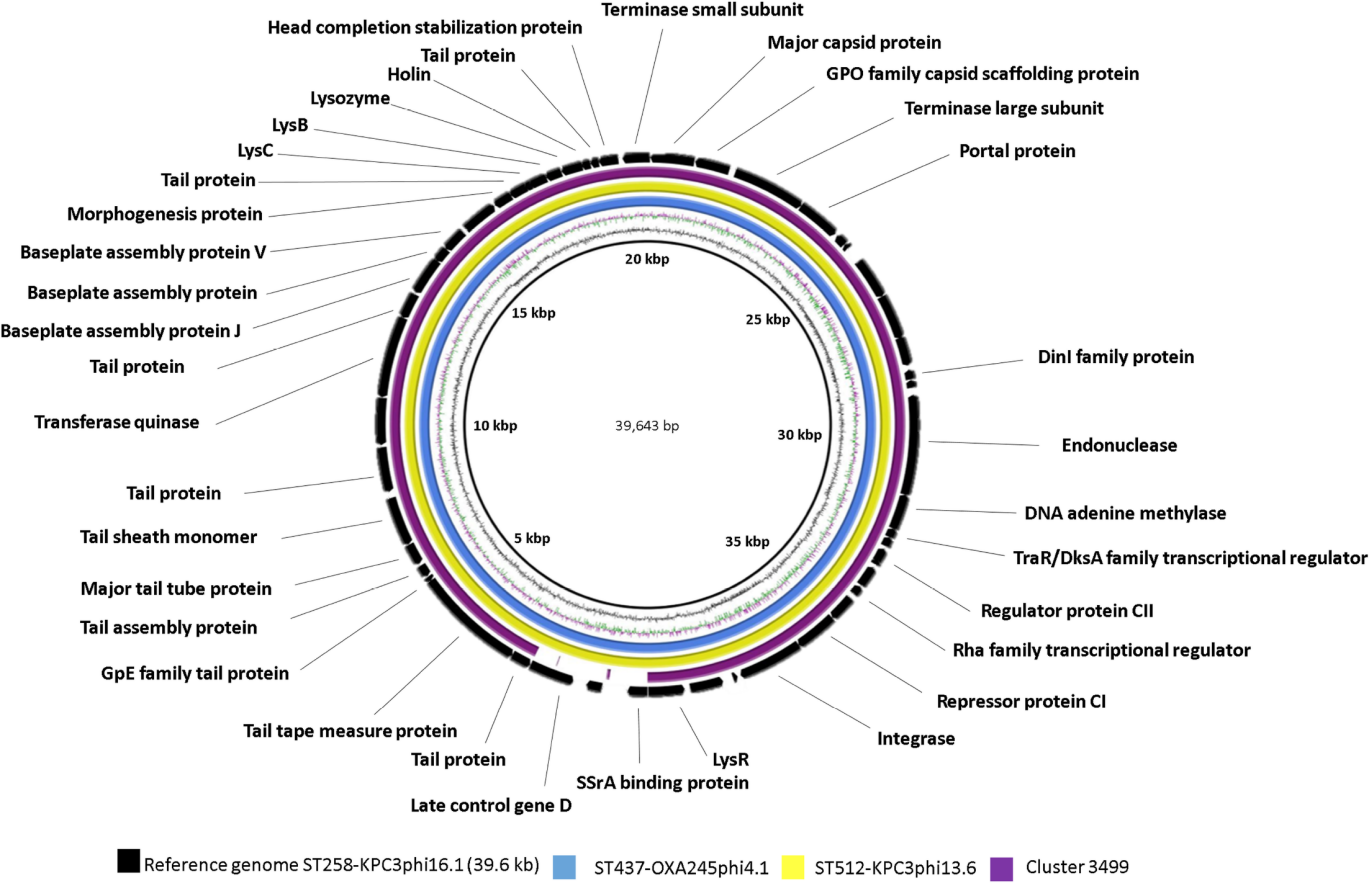
Comparative genomic analysis of *K. pneumoniae* temperate bacteriophages displaying the highest sequence identity with cluster 3499 (% query cover >92; % identity>99.9) of the MVP database [https://mvp.medgenius.info], constructed with the BRIG program. The sequence (ST258-KPC3phi16.1) is located on the innermost side and is indicated in black. The double ring adjacent to the reference sequence represents the GC content (black) and the GC skew (purple and green). The whitepart of the rings represents absent or divergent content relative to the unknown sequence. All prophages wereof size 39.6 kb (ST437-OXA245phi4.1 and ST512-KPC3phi13.6).

**Fig. 4.**
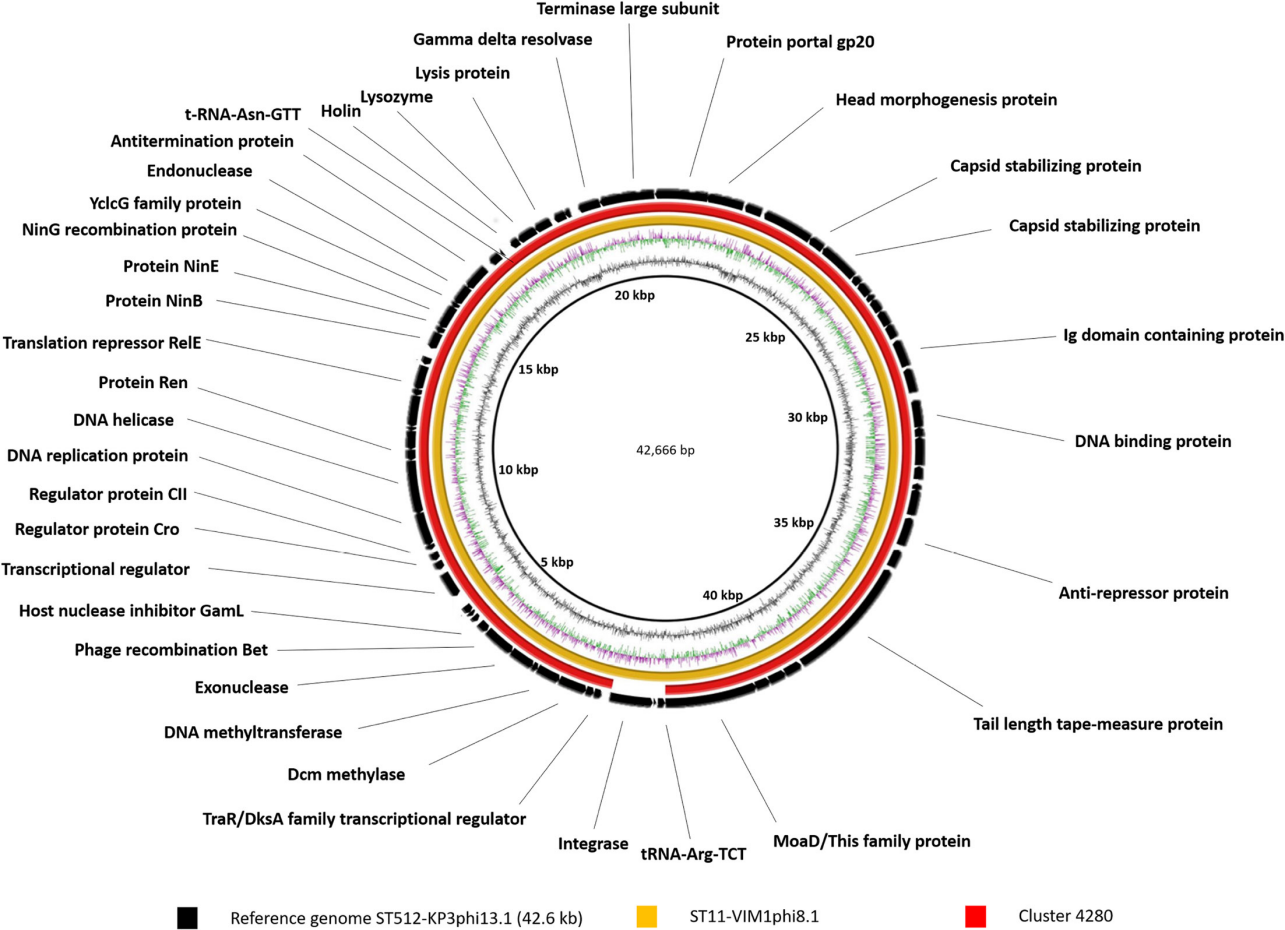
Comparative genomic analysis of *K. pneumoniae* temperate bacteriophages displaying the highest sequence identity with cluster 4280 (query cover >98.9 %; identity >99.9 %) from the MVP database (https://mvp.medgenius.info) analysed with the brig program. The sequence (ST512-KPC3phi13.1) is located on the innermost side and is indicated in black. The double ring adjacent to the reference sequence represents the G+C content (black) and the G+C skew (purple and green). The other rings are as indicated in the key. The white parts of the rings represent absent or divergent content relative to the unknown sequence. All prophages were of size 42.6 kb (ST11-VIM1phi8.1).

After annotation of the prophage genomes (see the Supplementary Material, available with the online version of this article) with rast, blastp and HHpred, it was evident that four of them (33.3, 36.1, 39.6 and 42.6 kb) were represented in different strains of *K. pneumoniae* (in seven, two, three and two strains, respectively) (Table 3). Comparative genomic analysis of these prophages was performed with the brig program according to their sequence similarities (Figs 1–4). Comparison of the sequences with different prophage sequences and clusters from the MVP database (see Table 3, Figs 1–4) showed that the 33.3 kb prophage genome sequences derived from different *K. pneumoniae* isolates were identical in multiple prophages (ST11-OXA245phi3.1, ST340-VIM1phi10.2, ST11-OXA48phi15.3 and ST258-KPC3phi16.2). The genome sequences also displayed a 100 % sequence identity with MVP database cluster 4762, which has the highest host range (67 bacterial strains) of all phage clusters included in the MVP database and is also known to infect *K. pneumoniae *, among other hosts (Fig. 1). Moreover, the 33.3 kb genomes displayed only partial sequence identity with genomes of the other two prophages (32.302 kb ST512-KPC3phi13.2 and 33.016 kb ST11-VIM1phi8.4), possibly due to their shorter size. We believe that prophage ST437-OXA245phi4.2, also belonging to the 33.3 kb cluster, is an incomplete prophage, as its genome is about half the size of that of the 33.3 kb genomes (18.281 kb versus 33.326 kb). The sequence of the 36.1 kb prophage genome was identical in two prophages (ST340-VIM1phi10.1 and ST11-OXA48phi15.1) and it was also identical to cluster 4901 (which showed a host range of 57 bacterial strains) (Fig. 2). The sequence of the 39.6 kb prophage was identical to the genomes of three prophages (ST437-OXA48phi4.1, ST512-KPC3phi13.6 and ST258-KPC3phi16.1). In addition, cluster 3499 (which showed a 19 strain host range) had partial sequence identity with the three prophages (Fig. 3). Finally, the sequence of the 42.6 kb temperate bacteriophage (prophage) (ST512-KPC3phi13.1) had an identical sequence in the prophage (ST11-VIM1phi8.1) and the cluster 4280 (Fig. 4).

**Table 3.** Comparison by sequence of prophage genomes and bacterial genomes

Prophage size (bp)*	Prophage		Bacterial strain		MPV international cluster‡
	**Name**	**Genome accession no.†**	**Name**	**Genome accession no.†**	
33 226	ST11-OXA245phi3.1	MK416009.1	ST11-OXA245	WRXH00000000	4762
	ST437-OXA245phi4.2	MK416012.1	ST437-OXA245	WRXG00000000	
	ST11-VIM1phi8.4	MK416020.1	ST11-VIM1	WRXC00000000	
	ST340-VIM1phi10.2	MK422454.1	ST340-VIM1	WRXA00000000	
	ST512-KPC3phi13.2	MK422449.1	ST512-KPC3	WRWY00000000	
	ST11-OXA48phi15.3	MK433580.1	ST11-OXA48	WRWW00000000	
	ST258-KPC3phi16.2	MK433582.1	ST258-KPC3	WRWV00000000	
36 124	ST340-VIM1phi10.1	MK422455.1	ST340-VIM1	WRXA00000000	4901
	ST11-OXA48phi15.1	MK433579.1	ST11-OXA48	WRWW00000000	
39 643	ST437-OXA48phi4.1	MK416011.1	ST437-OXA48	WRXG00000000	3499
	ST512-KPC3phi13.6	MK433577.1	ST512-KPC3	WRWY00000000	
	ST258-KPC3phi16.1	MK4333581.1	ST258-KPC3	WRWV00000000	
42 666	ST11-VIM1phi8.1	MK448233.1	ST11-VIM1	WRXC00000000	4280
	ST512-KPC3phi13.1	MK448235.1	ST512-KPC3	WRWY00000000	

*Prophage genomes located in the genomes of different *K. pneumoniae* clinical strains have been placed into four size groups (33.3, 36.1, 39.6 and 42.6 kb).

†Accession numbers of genomes from BioProject accession no. PRJNA565865.

‡MVP international clusters 4762, 4901, 3499 and 4280 from the MVP database (https://mvp.medgenius.info).

### Integration sites of prophages

In all prophages, the attachment sites (*attL* and *attR*) were identified by phaster. Moreover, by studying the integration sites of the prophages, we observed that these were integrated at various sites, as detailed in the following text. (i) Fourteen prophages were integrated before or after intact host tRNA (ST405-OXA48phi1.2, ST405-OXA48phi1.3, ST15-VIM1phi2, ST437-OXA245phi4.1, ST101-KPC2phi6.1, ST101-KPC2phi6.2, ST147-VIM1phi7.2, ST11-VIM1phi8.1, ST13-OXA48phi12.1, ST13-OXA48phi12.2, ST13-OXA48phi12.5, ST512-KPC3phi13.1, ST512-KPC3phi13.6 and ST258-KPC3phi16.1), with tRNA-arg being the most common host tRNA present before prophages in six cases. (ii) Seven prophages were integrated between the TerT transcriptional regulator intact genes and transporter intact genes (all bacteriophages 33.3 kb: ST11-OXA245phi3.1, ST437-OXA245phi4.2, ST11-VIM1phi8.4, ST340-VIM1phi10.2, ST512-KPC3phi13.2, ST11-OXA48phi15.3 and ST258-KPC3phi16.2), integration of the prophages did not truncate these genes. (iii) Four prophages were integrated next to the host transcriptional regulator (ST405-OXA48phi1.1, ST11-OXA245phi3.2, ST16-OXA48phi5.2 and ST846-OXA48phi9.1). In some cases (ST16-OXA48phi5.2, ST846-OXA48phi9.1), the integration of the prophage involved the disruption of the adjacent gene. (iv) Four prophages were integrated within the Sap*ABCDEF* operon that encodes an ATP-binding cassette (ABC) transporter, more precisely between *sapB* and *sapC* intact gene (ST16-OXA48phi5.1, ST11-VIM1phi8.2, ST846-OXA48phi9.2 and ST974-OXA48phi18). (v) Five prophages were integrated immediately after an intact protease (ST16-OXA48phi5.3, ST11-VIM1phi8.3, ST340-VIM1phi10.1, ST512-KPC3phi13.5 and ST11-OXA48phi15.1). (vi) Five prophages were integrated next to a protein of unknown function (ST101-KPC2phi6.3, ST147-VIM1phi7.1, ST13-OXA48phi12.3, ST13-OXA48phi12.4 and ST15-OXA48phi14). In the case of ST15-OXA48phi14, the phage integration truncated the protein of unknown function. (vii) One prophage was integrated after a sensor domain-containing diguanylate cyclase, truncating the latter (ST16-OXA48phi5.4).

### Phylogenetic study

**Fig. 5.**
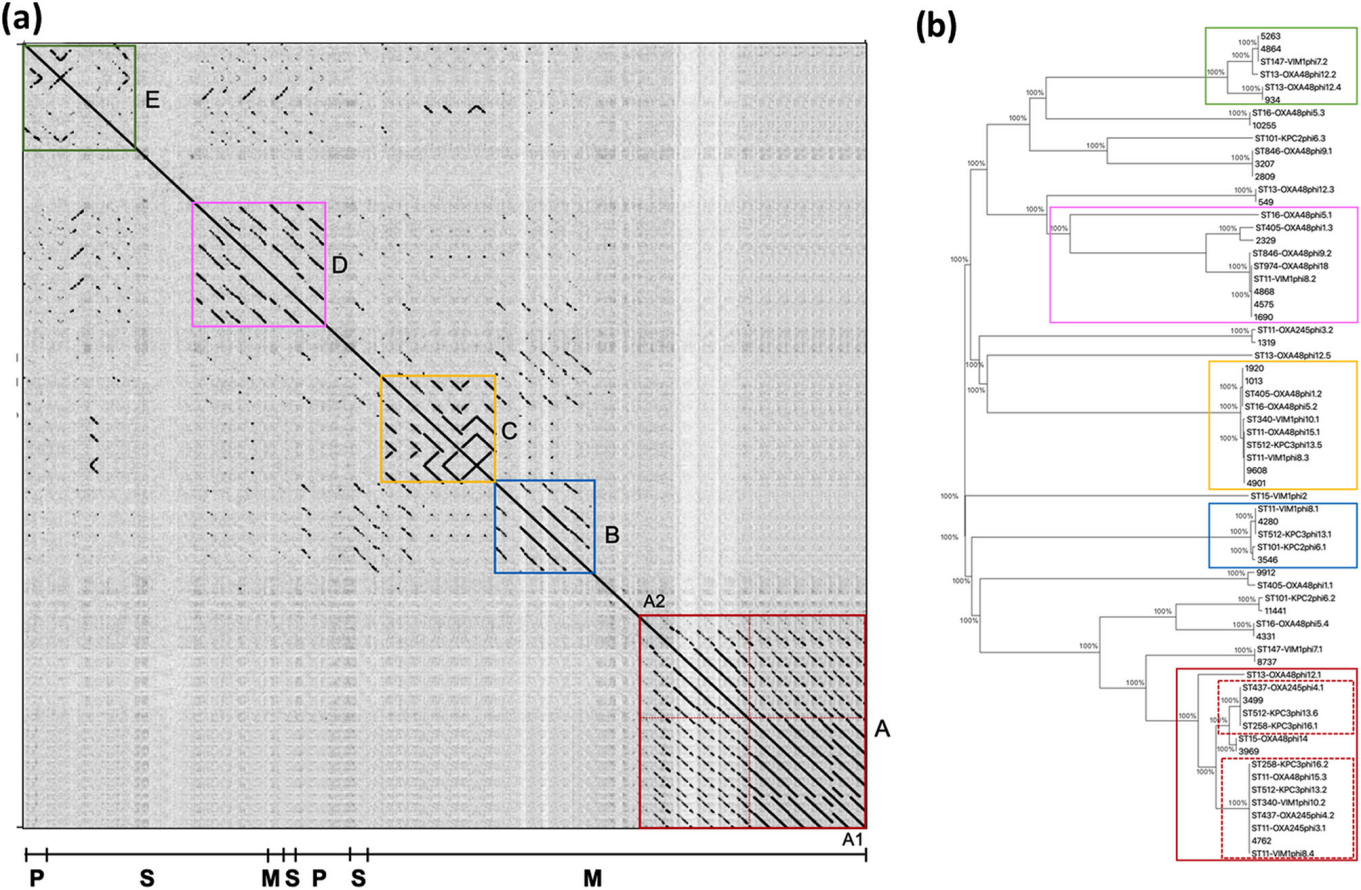
(a) Dot plot alignment of nucleotide sequences from 40 *K. pneumoniae* temperate bacteriophage genomes inferred using the gepard tool. For this purpose, a fasta file was constructed with all sequences. Black diagonal lines parallel to the main diagonal indicate strong sequence similarity, while grey lines indicate lower sequence similarity. The differently coloured boxes illustrate the assignment of clusters A (A1; A2), B, C, D and E, and are represented in red, blue, yellow, pink and green, respectively. The bacteriophage families are shown on the horizontal axis (M, *Myoviridae*; P, *Podoviridae*; S, *Siphoviridae*). (b) The evolutionary history of the large terminase subunit protein of 40 *K. pneumoniae* temperate bacteriophages and the representative MVP clusters were inferred using the neighbour-joining method. The percentage of replicate trees in which the associated taxa clustered together in the bootstrap test (1000 replicates) is shown next to the branches. The evolutionary distances were computed using the number of differences method. All positions containing gaps and missing data were eliminated. Evolutionary analyses were conducted in mega x [35].

To observe the phylogenetic relationships between the temperate bacteriophages under study and the clusters included in the MVP database, we reconstructed a neighbour-joining tree by the dot plot alignment of nucleotide sequences, constructed from a fasta file with all genomes of the 40 prophages studied with the gepard program. This enabled us to define five clusters: A, B, C, D and E (Fig. 5a). Cluster A was subdivided because it included two different groups of temperate bacteriophages (A1 and A2).

Moreover, we reconstructed a neighbour-joining tree (Fig. 5b) with a packaging protein, the terminase large subunit, in the tree with bootstrap values (100 %) [41]. The four temperate bacteriophages, which were included in different strains considered in the study (33.3, 36.1, 39.6 and 42.6 kb, together with their international clusters from MVP database, 4762, 4901, 3499 and 4280), were included in clusters and subclusters: A1, C, A2 and B, respectively (Table 3, Fig. 5b).

### TEM studies

**Fig. 6.**
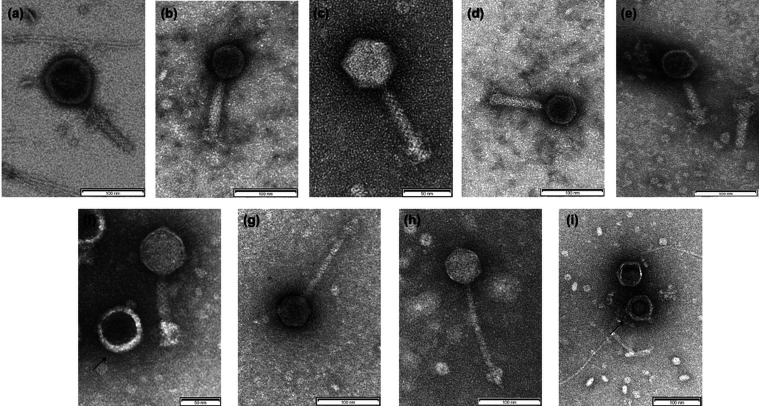
TEM images showing the different families of prophages present in different clusters. (a–e) The family *Myoviridae* obtained from ST11-OXA245phi3.1, ST13-OXA48phi12.1, ST101-KPC2phi6.1, ST405-OXA48phi1.2 and ST846-OXA48phi9.2 prophages belonging to A1, A2, B, C and D clusters, respectively. (f and i) The family *Podoviridae* obtained from ST11-VIM1phi8.2 and ST147-VIM1phi7.2 prophages belonging to D and E clusters, respectively. (g and h) The family *Siphoviridae* obtained from ST974-OXA48phi18 and ST13-OXA48phi 12.2 prophages belonging to D and E clusters, respectively.

TEM images revealed the presence of the three families of bacteriophages in the order *Caudovirales*. Representative images of the bacteriophages in each cluster are shown in Fig. 6. The typical morphology of the different families was observed, including a long, rigid tail in the *Myoviridae* bacteriophages (Fig. 6a–e), a long, flexible tail in the *Siphoviridae* bacteriophages (Fig. 6g, h) and a small tail in the *Podoviridae* bacteriophages (Fig. 6f, i).

### Induced bacteriophage mix spot test

The lytic potential of induced bacteriophages mixes with one phage representative of each cluster (previously observed by TEM) was assayed by the spot test in all the isolates of *K. pneumoniae* (Table 4). A spot was observed in one strain for all the bacteriophage mixes almost. The induced bacteriophage mix 3 derived from the strain ST101-KPC2, which harbours ST101-KPC2phi6.1 (48 131 bp; cluster B), ST101-KPC2phi6.2 (11 454 bp; not included in any cluster) and ST101-KPC2phi6.3 (43 942 bp; not included in any cluster), was able to produce a halo with five clinical isolates. Despite a large amount of halo, it is not possible to know whether it is due to the lytic capacity of bacteriophages or due to the phenomenon of lysis from without.

**Table 4.** Induced bacteriophage mix spot test Spot test assay of seven induced bacteriophage mixes isolated from seven clinical *K. pneumoniae* isolates. The infectivity of these cocktails was tested in the 16 *K. pneumoniae* strains studied. Bacteriophage mix 1, induced from ST405-OXA48, is composed of ST405-OXA48phi1.1, ST405-OXA48phi1.2 and ST405-OXA48phi1.3; bacteriophage mix 2, induced from ST11-OXA245, is composed of ST11-OXA245phi3.1 and ST11-OXA245phi3.2; bacteriophage mix 3, induced from ST101-KPC2, contains the bacteriophages ST101-KPC2phi6.1, ST101-KPC2phi6.2 and ST101-KPC2phi6.3; bacteriophage mix 4, induced from ST147-VIM1, contains ST147-VIM1phi7.1 and ST147phi7.2; bacteriophage mix 5, induced from ST11-VIM1, is composed of ST11-VIM1phi8.1, ST11-VIM1phi8.2, ST11-VIM1phi8.3 and ST11-VIM1phi8.4; bacteriophage mix 6, induced from ST846-OXA48, contains ST846-OXA48phi9.1 and ST846-OXA48phi9.2; and bacteriophage mix 7, induced from ST13-OXA48, contains ST13-OXA48phi12.1, ST13-OXA48phi12.2, ST13-OXA48phi12.3, ST13-OXA48phi12.4 and ST13-OXA48phi12.5. The induced bacteriophage mix 3 was able to produce halos in the highest number of strains, causing a halo in five different strains in five clinical isolates. −, Absence of lysis plaques; +, presence of lysis plaques.

Strain	Control (SM buffer)	Bacterial bacteriophage mix						
**1**	**2**	**3**	**4**	**5**	**6**	**7**
ST405-OXA48	−	+	−	+	−	−	−	+
ST15-VIM1	−	−	−	−	−	−	−	−
ST11-OXA245	−	−	−	−	−	−	−	−
ST437-OXA245	−	−	−	+	−	−	−	−
ST16-OXA48	−	−	−	+	−	−	−	−
ST101-KPC2	−	−	−	−	−	−	−	−
ST147-VIM1	−	−	−	−	−	−	−	−
ST11-VIM1	−	−	+	−	+	−	−	−
ST846-OXA48	−	−	−	−	−	−	−	−
ST340-VIM1	−	−	−	−	−	−	−	−
ST13-OXA48	−	−	−	−	−	−	−	−
ST512-KPC3	−	−	−	+	−	+	+	+
ST15-OXA48	−	−	−	−	−	−	−	−
ST11-OXA48	−	+	+	−	+	+	+	+
ST258-KPC3	−	−	−	+	−	−	−	−
ST974-OXA48	−	−	−	−	−	−	−	−

### Annotation of the prophages

**Fig. 7.**
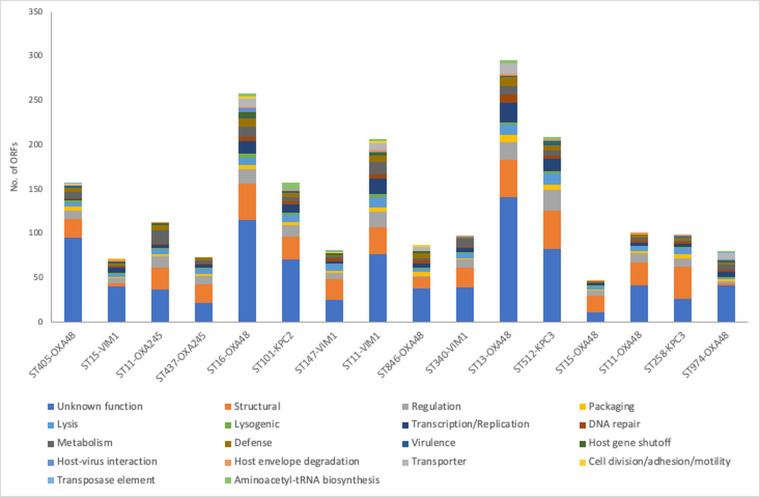
Numbers of ORFs carried by the prophages of each strain analysed, with associated functional categories.

Using rast, psi-blast and HHprep tools, we obtained functional information for 59.7 % of the prophage proteins analysed (Fig. 7, Supplementary Material).

The most abundant functional category found in the prophage genomes involved genes that constitute the minimum basic unit (universally presented genetic markers) of a *Caudovirales* bacteriophage, i.e. the genes related to structure and packaging, lysis, lysogenesis, transcription, replication and regulation (Fig. 7). One of the most important functions of the bacteriophage tail, and more precisely of the tail fibre and the baseplate protein, is to recognize the host surface receptors. In addition to these structural genes, the minimum unit of a bacteriophage was also composed of genes involved in packaging. The packaging machine of most bacteriophages contains two proteins called the terminase small subunit and the terminase large subunit [34]. In this study, the terminase large subunit (approx. 1700 bp) was identified and annotated in all genomes of the temperate bacteriophages; however, the same did not occur with the small subunit (approx. 600 bp), which was only identified and annotated in 28 temperate bacteriophage genomes.

**Fig. 8.**
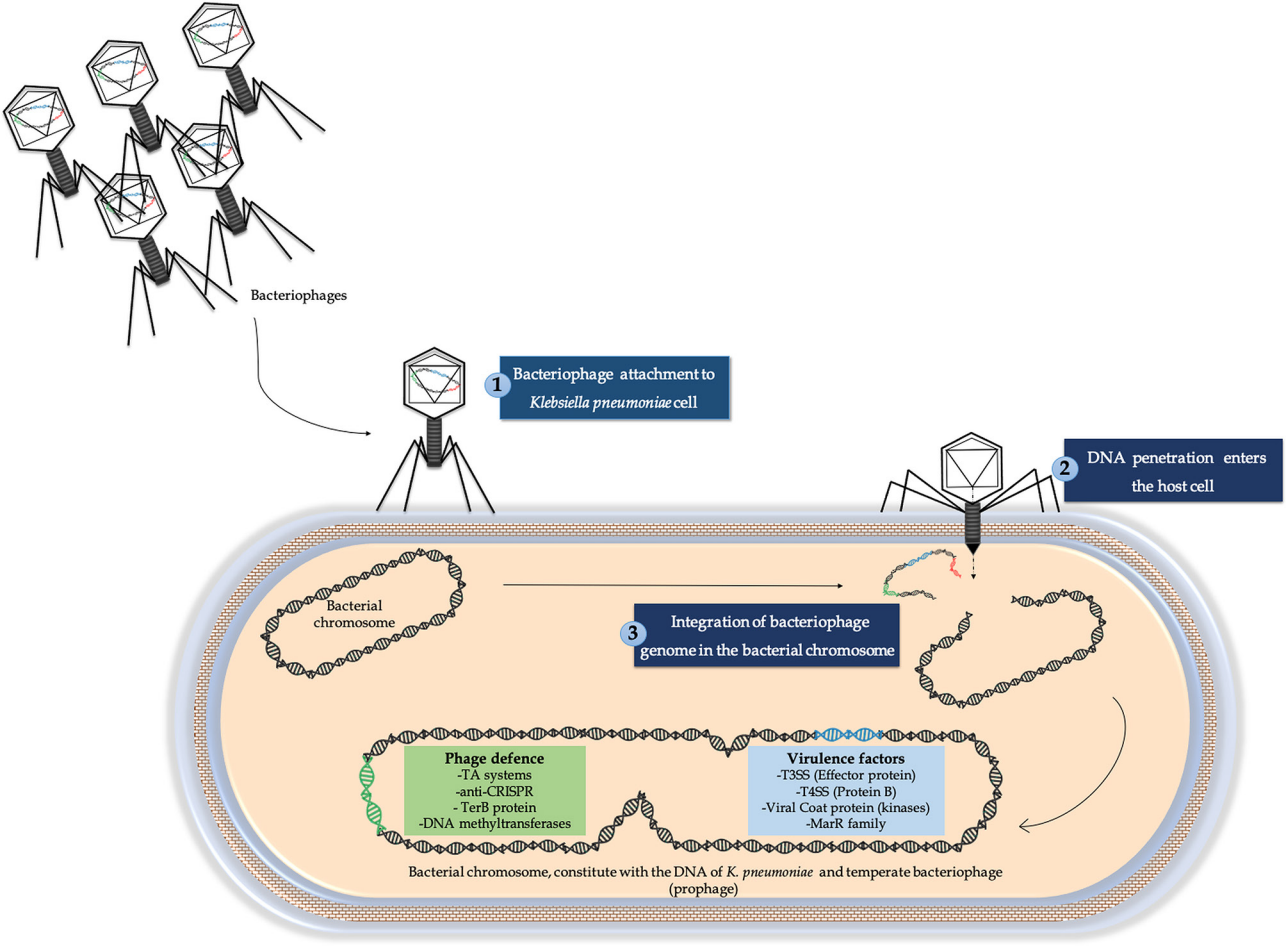
Illustration representative of transmission of virulence factor and phage defence genes by prophages in the bacterial chromosome of the *K. pneumoniae* clinical isolates considered in this study.

We also detected lysis genes involved in the lysis of the bacterial cell, such as the holin gene and lysozyme-encoding gene, in all viral genomes [42]. However, we did not consistently find lysogenic genes, such as integrase, in all of the prophages studied. In fact, 40.47 % of the studied prophages lacked integrase. Intending to study the insertion site of all the bacteriophages included in this study, we used the blastn tool to identify the region of the bacterial genome where this integration took place. This analysis revealed that the integrase was located upstream from what phaster considered to be the initial site in those bacteriophages that appeared to lack integrase, because this program is not able to identify sequences from two different contigs. In addition, the prophage genomes were also composed of genes (ORFs) involved in the phage–host interaction: virulence factors and phage defence genes (Fig. 8) [42].

The following virulence factors were identified in the collection of temperate bacteriophages of the *K. pneumoniae* clinical strains: (i) invasion-associated protein B T4SS (type IV secretion system) [43] (GenBank accession number QBQ71533.1; *E* value 1.60×10^−11^) from prophage ST512-KPC3phi13.1; (ii) viral coat protein (transferase-kinase) [44, 45] (GenBank accession numbers QBP27909.1, QBP08028.1 and QBP27756.1; *E* values 4×10^−37^, 4×10^−37^ and 4×10^−37^, respectively) in prophages ST258-KPC3phi16.1, ST437-OXA245phi4.1 and ST512-KPC3phi13.6, respectively; and (iii) the MarR family of transcriptional regulators [46] involved in regulating cellular processes such as antibiotic resistance and the expression of virulence factors in several members of the family *Enterobacteriaceae *. This MarR-like protein regulator (GenBank accession numbers QEA09493.1, QBP27467.1, QBP28293.1, QBQ71610.1, QBP28507.1 and QBP28244.1: *E* values 2.00×10^−68^, 4.60×10^−12^, 9.00×10^−69^, 1.6×10^−14^, 9.00×10^−69^ and 3.00×10^−68^, respectively) was located in prophages ST13-OXA48phi12.5, ST13-OXA48phi12.3, ST16-OXA48phi5.2, ST101-KPC2phi6.3, ST405-OXA48phi1.3, ST11-VIM1phi8.2 and ST15-VIM1phi2.1.

Regarding phage defence against attack from bacteria as well as other viruses, we highlight the following. (i) The presence of four TA modules, two of which had partial sequence identity with RelBE-like TA proteins [47, 48] (GenBank accession numbers QBP08163.1 and QBP08164.1, *E* value 8.3×10^−4^; and GenBank accession numbers QBP07972.1 and QBP07973.1, *E* value 1.2×10^−7^) located in prophages ST405-OXA48phi1.2 and ST16-OXA48phi5.3, respectively. The other two TA systems showed sequence identity with HigBA-like TA modules located in the prophages ST11-VIM1phi8.3 and ST846-OXA48phi9.2 [48] (GenBank accession numbers QBP07854.1 and QBP07855.1, *E* value 5.4×10^−4^; and GenBank accession numbers QBP07684.1 and QBP07685.1, *E* value 1.2×10^−8^). (ii) The presence of two CRISPR-associated endoribonuclease Cas2 located in prophages ST512-KPC3phi13.6 and ST437-OXA245phi4.1 (GenBank accession numbers QBP27775.1 and QBP08047.1; *E* values 4.9×10^-14^ and 6.2×10^-14^, respectively) [48]. (iii) The presence of one putative anti-CRISPR/Cas9 protein, AcrIIC3-like, located in prophage ST846-OXA48phi9.2 (GenBank accession number QBP0798.1; *E* value 3.7×10^−8^) [48]. (iv) The presence of TerB protein [49] (GenBank accession number QBP27498.1; *E* value 6.00×10^−93^) in prophage ST13-OXA48phi12.3 from the operon *terZABCDEF*, which confers tellurite resistance and has been related to the inhibition of bacteriophages. (v) The presence of 32 ORFs of methyltransferase proteins [44] in 25 prophages from these *K. pneumoniae* clinical isolates (Table 5).

**Table 5.** GenBank accession numbers and protein IDs for the predicted methyltransferase ORFs carried by the prophages of the *K. pneumoniae* clinical isolates

Prophage	GenBank accession no.	Protein ID	*E* value
ST405-OXA48phi1.2	MK416007.1	QBP08186.1	2.00E–164
ST11-OXA245phi3.1	MK416009.1	QBP08076.1	0.00E+00
ST11-OXA245phi3.2	MK416010.1	QBQ71801.1	3.00E–147
ST437-OXA245phi4.1	MK416011.1	QBP08055.1	0.00E+00
ST437-OXA245phi4.2	MK416012.1	QBQ71739.1	0.00E+00
ST16-OXA48phi5.1	MK416013.1	QBQ71677.1	2.00E–52
		QBQ71678.1	5.00E–54
ST16-OXA48phi5.2	MK448230.1	QBP28277.1	0.00E+00
		QBP28278.1	2.00E–126
ST16-OXA48phi5.4	MK416015.1	QBP07929.1	0.00E+00
ST101-KPC2phi6.1	MK448231.1	QBP28349.1	0.00E+00
		QBP28350.1	5.00E–160
ST101-KPC2phi6.3	MK416017.1	QBQ71614.1	0.00E+00
ST147-VIM1phi7.2	MK448232.1	QBP28455.1	0.00E+00
ST11-VIM1phi8.1	MK448233.1	QBQ71410.1	0.00E+00
		QBQ71411.1	5.00E–160
ST11-VIM1phi8.4	MK416020.1	QBP07832.1	0.00E+00
ST846-OXA48phi9.2	MK416022.1	QBP07693.1	3.00E–117
ST340-VIM1phi10.2	MK422454.1	QBP27648.1	0.00E+00
ST13-OXA48phi12.1	MK422453.1	QBP27632.1	0.00E+00
ST13-OXA48phi12.2	MK422452.1	QBP27559.1	7.00E–160
ST13-OXA48phi12.3	MK422451.1	QBP27503.1	2.00E–141
ST13-OXA48phi12.4	MK422450.1	QBQ71947.1	2.00E–116
ST13-OXA48phi12.5	MK714353.1	QEA09480.1	0.00E+00
		QEA09481.1	1.00E–159
ST512-KPC3phi13.1	MK448235.1	QBQ71476.1	0.00E+00
		QBQ71477.1	5.00E–160
ST512-KPC3phi13.2	MK422449.1	QBP27409.1	0.00E+00
ST512-KPC3phi13.6	MK433577.1	QBP27783.1	0.00E+00
ST15-OXA48phi14	MK433578.1	QBQ71986.1	0.00E+00
ST258-KPC3phi16.1	MK433581.1	QBP27938.1	5.00E–179
ST258-KPC3phi16.2	MK433582.1	QBP27988.1	0.00E+00

Finally, we observed the presence of tRNA in the genome of eight prophages (ST16-OXA48phi5.2, ST101-KPC2phi6.1, ST147-VIM1phi7.1, ST11-VIM1phi8.1, ST13-OXA48phi12.1, ST13- OXA48phi12.5, ST512-KPC3phi13.1 and ST974-OXA48). In most cases (ST16-OXA48phi5.2, ST101-KPC2phi6.1, ST11-VIM1phi8.1, ST13-OXA48phi12.5, ST512-KPC3phi13.1 and ST974-OXA48), the tRNAs were located between the phage antitermination protein and lysis-related proteins, such as holin. By contrast, in prophages ST147-VIM1phi7.1 and ST13-OXA48phi12.5, the tRNAs were located between the G/U mismatch-specific uracil DNA glycosylase and a hypothetical protein [50].

## Discussion

In this study, we identified and annotated the genomes of 40 prophages (mainly belonging to the order *Caudovirales* family *Myoviridae*) from 16 clinical strains of carbapenemase-producing *K. pneumoniae* and determined their phylogenetic relationships. According to the available data about the size of the phage genomes, we observed diverse sizes of temperate bacteriophages, ranging from 11.445 kb (prophage ST101-KPC2phi6.2) to 84.199 kb (prophage ST13-OXA48phi12.3) and corresponding to the size of dsDNA viruses (i.e. 18 to 500 kb) [51]. Interestingly, prophages of sizes 33.3, 36.1, 39.6 and 42.6** **kb were present in several clinical strains of *K. pneumoniae *, possibly indicating important roles in these isolates. Moreover, these prophages had partial sequence identity with international bacteriophage clusters 4762, 4901, 3499 and 4280 (MVP database), respectively, which are the most prevalent clusters in clinical strains of *K. pneumoniae *. Interestingly, these temperate bacteriophages were included by phylogenetic relationships in three main clusters (A, B and C). These findings indicate the high frequency of temperate bacteriophages in clinical populations of *K. pneumoniae* [52, 53], as well as in other clinical pathogens such as *Acinetobacter baumannii* [8] and *Pseudomonas aeruginosa* [9, 54].

Interestingly, analysis of spot tests revealed that the most frequent temperate bacteriophages (33.3, 36.1, 39.6 and 42.6 kb) do not produce halos in the highest number of strains. This is probably due to the superinfection exclusion systems promoted by the prophages in the bacterial genome. Thus, the bacteria become immune to subsequent infection by other bacteriophages that are the same or very similar to the integrated prophages [55].

The integration of the bacteriophages into the bacterial genome is a crucial step in the lysogenic cycle [56]. This event is mediated by the integrase protein, a DNA recombinase encoded by bacteriophages, at a specific bacterial genome attachment site (*attB*), which is identical to an attachment site (*attP*) of the bacteriophage genome [56]. According to previous reports, there have been indications that prophages are not randomly distributed in genomes. Indeed, it has been observed that those prophages that encode tyrosine integrase are usually integrated next to a host tRNA [57]. One possible explanation for this finding is the affinity that the temperate bacteriophage may have for palindromic structures, enabling integration [57]. These data corroborate our findings, whereby 35 % of the prophages under study were integrated close to host tRNA, specifically tRNA-ARG, which coincides with the findings of Roszniowski *et al*. for a *Burkholderia cenocepacia* prophage sequence [58]. Additionally, the subsequent integration sites that we identified are also consistent with those most frequently observed by these researchers (ABC transporter genes and transcriptional regulators) [58].

Most of the genes that encode bacteriophages (50–80 %) lack a described function to date and, therefore, are currently deposited in public databases, such as the NCBI, as hypothetical proteins [59–61]. Thus, several studies have revealed a high percentage of hypothetical proteins in the genomes of specific bacteriophages of *Salmonella* [36], *K. pneumoniae* [62] and *Escherichia coli* [63]. In the present study, we predicted the function of 59.7 % of proteins, by using different bioinformatics tools. Identification of protein functions is a challenge that must be addressed to improve knowledge of bacteriophages and the level of security of their applications [36]. It should be noted that in other studies a better understanding of the bacteriophage's protein function allowed bacteriophage engineering to be undertaken. In fact, knowledge of bacteriophage regulatory proteins has allowed the conversion of a lysogenic bacteriophage into a lytic one in *A. baumannii *, which presented activity against various clinical strains of *A. baumannii* [64]. In addition, a cocktail of natural lytic bacteriophages along with engineered bacteriophages has recently been successfully used to treat an infection caused by a drug-resistant *Mycobacterium abscessus* strain in a patient with cystic fibrosis [65].

We subsequently observed that the next most abundant genes in the prophage genomes were those that constitute the minimum unit of a *Caudovirales* bacteriophage, i.e. genes related to structure, packaging, lysis, lysogenesis, transcription, replication and regulation [66, 67]. In the present study, we predicted virulence factors belonging to secretion systems such as T4SS (type IV secretion system) (involved in bacterial competence) [68], viral coat proteins [69] and the MarR family regulator of efflux pumps (which are associated with virulence factors in many bacteria [46]). For example, SlyA regulates *Salmonella* pathogenicity island-2 genes and contributes to resistance to oxidative stress, bacterial survival within macrophages and also bacterial survival in infection models. In another example, RovA is a member of the MarR/SlyA family, which regulates expression of *inv* (an adhesion and invasion factor) in the enteric pathogens *Yersinia enterocolitica* and *Yersinia pseudotuberculosis,* as well as expression of the *psa* locus of *Yersinia pestis *, the causative agent of bubonic and pneumonic plague. Members of this transcriptional regulator family are also thought to be important for the adaptation of *K. pneumoniae* in the mammalian host [70].

Interestingly, several mechanisms associated with phage defence against other viruses or bacteria have been detected. We must highlight the prediction of modules of TA systems in the genome of some prophages from this study. TA systems are small modules consisting of a stable toxin and its unstable cognate antitoxin [71]. These systems are involved in abortive infection (bacteriophage immunity through altruistic suicide), which not only protects bacteria from bacteriophage infection in the cultures grown, but also affects the use of bacteriophage therapy [48]. The use of bioinformatics tools has led to the detection of four TA modules. Two of these modules displayed partial sequence identity with RelBE-like proteins, and the other two displayed partial sequence identity with HigBA TA proteins [47, 48].

We also detected a putative anti-CRISPR/Cas9 protein, AcrIIC3. The CRISPR/Cas system is known to play an important role in protecting bacteria against invasion by bacteriophages and other mobile genetic elements. Nevertheless, bacteriophages have evolved different means of evading CRISPR/Cas defence systems, such as point mutations and the actions of small proteins that directly interact with the CRISPR/Cas system and shut it down. The first anti-CRISPR protein was discovered in 2013, in *P. aeruginosa* prophages [72, 73], and since then multiple anti-CRISPR/Cas proteins have been discovered in bacteriophages of various bacterial species, e.g. *Moraxella bovoculi *, *Sulfolobus islandicus*, *Listeria monocytogenes* and *Streptococcus thermophilus* [74]. The anti-CRISPR/Cas9 AcrIIC3 was discovered in *Neisseria meningitis* and acts by inhibiting the Cas9 protein, blocking the DNA loading step through binding to a non-conserved surface of the HNH domain and interacting with the rec lobe of Cas9 leading to dimerization of the ArcII3-Cas9 complex [75, 76]. We also detected a CRISPR-associated endoribonuclease Cas2 in the genomes of two prophages. Bacteriophages are thought to use such proteins to evade host immunity in an as yet uncharacterized way [77].

Also related to phage infection immunity, we found the TerB protein, despite it being involved in resistance to tellurite when it is part of *terZABCDEF* operon in bacterial pathogens such as *E. coli* and *K. pneumoniae* [78]. Furthermore, TerB has been found to be involved in resistance to infection by various bacteriophages, such as T1 and T5, a mechanism known as phage inhibition (Phi), as well as related with resistance to pore-forming colicins (PacB) [79]. Finally, numerous predicted methyltransferase enzymes were located in 62.5 % of the prophages from *K. pneumoniae* clinical strains, and were reported to be involved in protecting the viral genome against the host restriction enzymes from the bacteria [53, 80].

### Conclusion

This study characterized 40 prophages (order *Caudovirales*) in the genomes of 16 clinical strains of *K. pneumoniae* belonging to different STs (MLST). The analysis revealed the presence of four size groups of prophages (33.3, 36.1, 39.6 and 42.6 kb), whose sequences were similar to those of the international host-bacteriophage clusters registered in the MVP database, 4762, 4901, 3499 and 4280, respectively. Moreover, these 33.3, 36.1, 39.6 and 42.6 kb prophages were included in three main clusters (A, B and C) by phylogenetic relationships. The annotation of prophages has revealed 40.3 % of prophage genomes encoded genes of unknown function. However, this annotation has also revealed that the second most important group of genes constituted the minimum unit of *Caudovirales* bacteriophages. Interestingly, we observed virulence factors (compounds of secretion systems or regulators) and components of phage defence against bacteria and also against other bacteriophages (TA modules, anti-CRISPR/Cas9, TerB protein and methyltransferase proteins) in prophage genomes. Future lines of research should focus on obtaining more information about genes of unknown function to provide a better understanding of phage genomes for possible therapeutic applications of bacteriophages, such as phage-derived protein, as well as for engineering phages with activity against MDR pathogens.

## Data Bibliography

1. Forty-two bacteriophages isolated from 17 clinical strains of *Klebsiella*: genome sequencing and assembly (REIPI – Spanish Network for Research in Infectious Diseases). Bleriot I, Trastoy R, Blasco L, Fernández-Cuenca F, Ambroa A, Fernández-García L, Pacios O, Perez-Nadales E, Torre-Cisneros J, Oteo-Iglesias J, Navarro F, Miró E, Pascual A, Martínez-Martínez L, Tomás M. BioProject accession number PRJNA565865 (https://www.ncbi.nlm.nih.gov/bioproject/?term=PRJNA565865) (2019).

## Supplementary Data

Supplementary material 1
